# Donor-dependent variation of human umbilical cord blood mesenchymal stem cells in response to hypoxic preconditioning and amelioration of limb ischemia

**DOI:** 10.1038/s12276-017-0014-9

**Published:** 2018-04-20

**Authors:** Insung Kang, Byung-Chul Lee, Soon Won Choi, Jin Young Lee, Jae-Jun Kim, Bo-Eun Kim, Da-Hyun Kim, Seung Eun Lee, Nari Shin, Yoojin Seo, Hyung-Sik Kim, Dong-Ik Kim, Kyung-Sun Kang

**Affiliations:** 1 Adult Stem Cell Research Center, College of Veterinary Medicine, Seoul National University, Seoul, 08826 Republic of Korea; 2 Research Institute for Veterinary Medicine, College of Veterinary Medicine, Seoul National University, Seoul, 08826 Republic of Korea; 3 Pusan National University School of Medicine, Busan, 49241 Republic of Korea; 4 Biomedical Research Institute, Pusan National University Hospital, Busan, 49241 Republic of Korea; 5 Division of Vascular Surgery, Samsung Medical Center, Sungkyunkwan University School of Medicine, Seoul, 06351 Republic of Korea

## Abstract

With the rapidly growing demand for mesenchymal stem cell (MSC) therapy, numerous strategies using MSCs for different diseases have been studied and reported. Because of their immunosuppressive properties, MSCs are commonly used as an allogeneic treatment. However, for the many donors who could potentially be used, it is important to understand the capacity for therapeutic usage with donor-to-donor heterogeneity. In this study, we aimed to investigate MSCs as a promising therapeutic strategy for critical limb ischemia. We evaluated MSCs from two donors (#55 and #64) and analyzed the capacity for angiogenesis through in vivo and in vitro assays to compare the therapeutic effect between different donors. We emphasized the importance of intra-population heterogeneity of MSCs on therapeutic usage by evaluating the effects of hypoxia on activating cellular angiogenesis in MSCs. The precondition of hypoxia in MSCs is known to enhance therapeutic efficacy. Our study suggests that sensitivity to hypoxic conditions is different between cells originating from different donors, and this difference affects the contribution to angiogenesis. The bioinformatics analysis of different donors under hypoxic culture conditions identified intrinsic variability in gene expression patterns and suggests alternative potential genetic factors ANGPTL4, ADM, SLC2A3, and CDON as guaranteed general indicators for further stem cell therapy.

## Stem cell therapy: Finding the best cells

Stem cells from some donors have a stronger therapeutic effect than others, and can be identified by genetic markers. Adult or mesenchymal stem cells (MSCs), which can differentiate into many other cell types, show promise to treat many diseases. Kyung-Sun Kang at Seoul National University and co-workers investigated whether MSCs from some donors show better therapeutic efficacy than others. They tested MSCs from several donors in a mouse model simulating a stroke in a hindlimb, and measured regrowth of blood vessels. MSCs from one donor showed higher regenerative efficacy, triggering regrowth of many blood vessels and allowing the limb to be saved. Kang and co-workers used genetic analysis to identify genes that showed higher expression in these MSCs. This gene expression profile could be used to select MSCs with a higher regenerative capacity.

## Introduction

Peripheral artery disease (PAD) remains a leading cause of limb disability and loss, which is caused by critical limb ischemia^1^. Although the disease severely diminishes quality of life and has a great risk of amputation, there are currently only a few treatment options. Recently, several types of research in cell therapy reported that cells have the potential to re-vascularize the ischemic limb^2^. Preclinical cell therapy studies have demonstrated the improved regeneration of the vascular system in different experimental models with several types of cell applications through various injection routes^3,4^. However, in clinical trials, the cell therapies showed varied outcomes; some of them improved in revascularization and led to less amputation, while many other trials did not show any clinical benefits^5^.

Mesenchymal stem cells (MSCs), a promising candidate source for cell transplantation therapies for PAD, are well-known for their distinctive qualities, such as immunomodulation^6^, maintaining endogenous stem cell niches^7^ and their potential to stimulate angiogenesis^8^. Additionally, they have been reported to migrate and proliferate in response to the cytokines or chemokines released from the ischemic site^9^. Recent studies have focused on modifying MSCs to improve revascularization and understand the cell’s biological role and mode of action in angiogenesis^10^. Despite these efforts and accomplishments, the results of current preclinical studies and clinical trials suggest that a better alleviation strategy with MSC therapy is still needed.

One strongly suggested element is that there are individual differences in MSCs based on the variability from donor to donor^11^. To verify MSCs as a reliable cell source and establish MSC cell therapy for PAD, the strikingly variable behaviors among MSCs isolated from different donors must be understood. Recent studies addressing this issue have compared bone marrow MSCs from various donors and found significant differences in cell growth rates and alkaline phosphatase enzyme activity^12^. Differentiation capacity also showed contrasting results between cells from different donors, with distinguished osteogenic differentiation ability with different gene levels, and the adipocyte-specific gene expression varied as well^13^.

In this study, we examined the angiogenesis capacity of human umbilical cord blood-derived mesenchymal stem cells (hUCB-MSCs) in vitro and in vivo. We focused on comparing hUCB-MSCs isolated from different donors, and examined the capability for therapeutic efficacy for PAD. To highlight the fact that individual differences based on donor-specific cellular properties is crucial in the application of the cells, we optimized the culture conditions of hUCB-MSCs by incubating in hypoxic conditions for 1 day or 2 weeks and analyzed the change in revascularization. Moreover, genome-wide analysis of hUCB-MSCs between different donors demonstrated different therapeutic efficacy through genetic profiling.

## Materials and methods

### Isolation and culture of hUCB-MSCs

Entire experimental procedures involving hUCB-MSCs were conducted under approval of the Boramae Hospital Institutional Review Board (IRB) and the Seoul National University IRB (IRB No. 1608/001-021). Isolation and culture of hUCB-MSCs were previously described^7^. In brief, human cord blood samples were incubated with HetaSep solution (Stem Cell Technologies, Vancouver, Canada) at a ratio of 5:1 to remove red blood cells. Then, the supernatant was collected with Ficoll, and mononuclear cells were separated after centrifugation at 2500 r.p.m. for 20 min. The cells were washed twice in phosphate-buffered saline (PBS). Cell pellets were reconstituted and seeded in KSB-3 Complete media (Kangstem Biotech, Seoul, Republic of Korea) containing 10% fetal bovine serum (Gibco BRL, NY, USA) and antibiotics. After 3 days of stabilization, unattached cells were washed out, and isolated stem cells were maintained at 5% CO_2_ and 21% O_2_ for normoxic condition. For hypoxic culture, hUCB-MSCs were transferred to a hypoxic chamber containing 5% CO_2_ and 1% O_2_ gases for 24 h or 2 weeks.

### Immunocytochemistry

Cells cultured under normoxic and hypoxic conditions were washed in PBS and fixed with 4% paraformaldehyde (PFA) at room temperature for 10 min. The cells were permeabilized with 0.05% Triton X-100 solution at room temperature for 10 min and blocked with 5% normal goat serum (NGS) at room temperature for 1 h. Then, the cells were stained with specific primary antibodies against HIF-1α (Abcam, Cambridge, UK) for >12 h and incubated for 2 h with Alexa 488-labeled secondary antibody (1:1000; Molecular Probes, Eugene, OR, USA). The counterstaining of nuclei was conducted with DAPI.

### Characterization of hUCB-MSCs

The stem cell properties of hUCB-MSCs cultured under normoxic and hypoxic conditions were investigated in three different ways. CD marker expression was measured by flow cytometry. To verify the characteristic of MSCs, the cells were stained with FITC- or PE-conjugated antibodies specific for human CD11b, CD34, CD44, CD45, CD73, CD105, and HLA-DR (BD Biosciences, San Jose, CA, USA) and analyzed by a FACScalibur using Cell Quest software (BD Biosciences, San Jose, CA, USA). CPDL analysis was used to determine the self-renewal capacities of the cells. Cell proliferation levels in each passage were calculated through the formula, CPDL = ln (Nf/Ni) ln2, where Ni is the initial number of cells seeded, Nf is the final number of collected cells, and ln is the natural log. Then, hUCB-MSCs were differentiated into three adipogenic, osteogenic, and chondrogenic lineages. For adipogenic and osteogenic differentiation, the attached cells were cultured with manually made adipogenic or an osteogenic differentiation medium. After 2 weeks of induction, adipogenesis was determined by staining with Oil Red O for intracellular lipid accumulation, and osteogenesis was visualized with Alizarin Red S staining, which is specific for calcium. For chondrogenic differentiation, the pellet-type cells were incubated with chondrogenic differentiation medium (Lonza, Allendale, NJ, USA). After 3 weeks, the pellets were fixed, processed, embedded, and sliced into 3 μm sections. The sections were stained with toluidine blue following standard procedures. The detailed protocols for flow cytometry, CPDL analysis and differentiation assays were previously described^7^.

### Capillary-like tube formation assay

Transwell inserts with a 4 µm pore size were coated with growth factor-reduced Matrigel at 30 μl/well and then placed at 37 °C overnight to allow gel formation. HUVECs were suspended in the media and adjusted to 2 × 10^4^/ml. Cell suspension (500 μl) was added to each well of the 24-well plate coated with Matrigel. hUCB-MSCs were then added to transwells with 500 µl of cell suspension (1 × 10^3^/ml) in triplicate. Each cell group was incubated under normoxic conditions for 24 h. HUVEC tubes were examined using a light microscope. Several images of each well were acquired and analyzed by measuring total length of tubes and counting total branching points.

### Cell migration assay

HUVECs were suspended in media, and 500 µl of cell suspension (1 × 10^4^/ml) was added to transwell inserts (8 µm pore size). Then, 500 µl of MSC condition media was added to the lower wells. After 24 h of incubation, HUVECs that migrated to the underside of the membrane were fixed, and the remaining cells in the upper chamber were carefully swiped off by a cotton swab. The membranes of the transwells were stained in DAPI and sealed on slides. A confocal microscope was used to count the number of cells on the underside of the insert for each group. The mean number of cells for each group was then estimated by calculating the mean of the three replicates.

### Induction of the hindlimb ischemia model

All the animal experiments were carried out in accordance with the approved guidelines of the Seoul National University Institutional Animal Care and Use Committee (IACUC No. SNU-160318-5). Four-week-old male BALB/c nude mice (Orientbio, Sungnam, Republic of Korea) were used for inducing the hindlimb ischemia model (Five mice per group). After the mice were anesthetized, the femoral artery of the left hindlimb was ligated and excised. Before closing the incision, 1 × 10^6^ cells of hUCB-MSCs that were incubated under normoxic and hypoxic conditions were injected into the quadriceps muscle divided into 5 places.

### Functional scoring

HLI-induced mice were evaluated for functional recovery using the Tarlov scoring system and the ischemia scoring system^14^ described in Supplementary Table 1 and 2 on Days 1, 3, 7, 14, and 28.

### Histopathological evaluation

Collected muscle samples were fixed in 10% formalin, prepared in accordance with typical processing steps, including dehydration with ethanol, clearing with xylene and wax infiltration with paraffin. Paraffin-embedded blocks were sectioned to 5 μm thickness and stained with H&E or picrosirius red with nuclei staining (Direct Red 80 and Fast Green FCF; Sigma Aldrich, St. Louis, MO, USA). Neutrophil infiltration and the area of necrotic tissue were measured by H&E staining. The area of remaining muscle fibers and fibrotic tissue were determined by picrosirius red staining.

### Cytokine production

To determine the secretion level of various cytokines, culture supernatants were collected from the hypoxic- or normoxic-cultured cells. ELISA kits for PGE_2_ (R&D Systems, Minneapolis, MN, USA), PGI_2_ (Cusabio, Wuhan, China) and VEGF (RayBiotech, Norcross, GA, USA) were used according to the manufacturer’s protocols.

### mRNA sequencing analysis

Three normalization methods (RPKM, RLE, and TMM) were applied to the N#55, SH#55, LH#55, N#64, SH#64, and LH#64 samples. The suitability of each normalization method can be evaluated by calculating its coefficient of variation (CV), where the lower the CV value, the better the normalization. Of the three methods used, we selected the RPKM as an optimal normalization method because it has the lowest CV (0.6479). The trimmed mean of *M*-value (TMM; included in the edgeR Bioconductor package) was computed as the weighted mean of log ratios between a specified test and the reference, and the computation was performed after the exclusion of the most expressed genes and the genes with the highest log ratios^15^. Genes enriched in Gene Ontology (GO) function categories were classified by their biological processes, and class, using the Protein ANalysis THrough Evolutionary Relationships (PANTHER) Classification system^16^.

### Statistical analysis

The mean values of all results were expressed as the mean ± SD. Statistical analyses were conducted using Student’s 2-tailed *t*-test or one-way ANOVA followed by Bonferroni post hoc test for multiple group comparisons using GraphPad Prism version 5.0 (GraphPad Software, San Diego, CA, USA). Statistical significance was designated with an asterisk, as indicated in the figure legends.

## Results

### Physiological and differentiation properties showed high similarity between normoxia and hypoxia-induced hUCB-MSCs

**Fig. 1:**
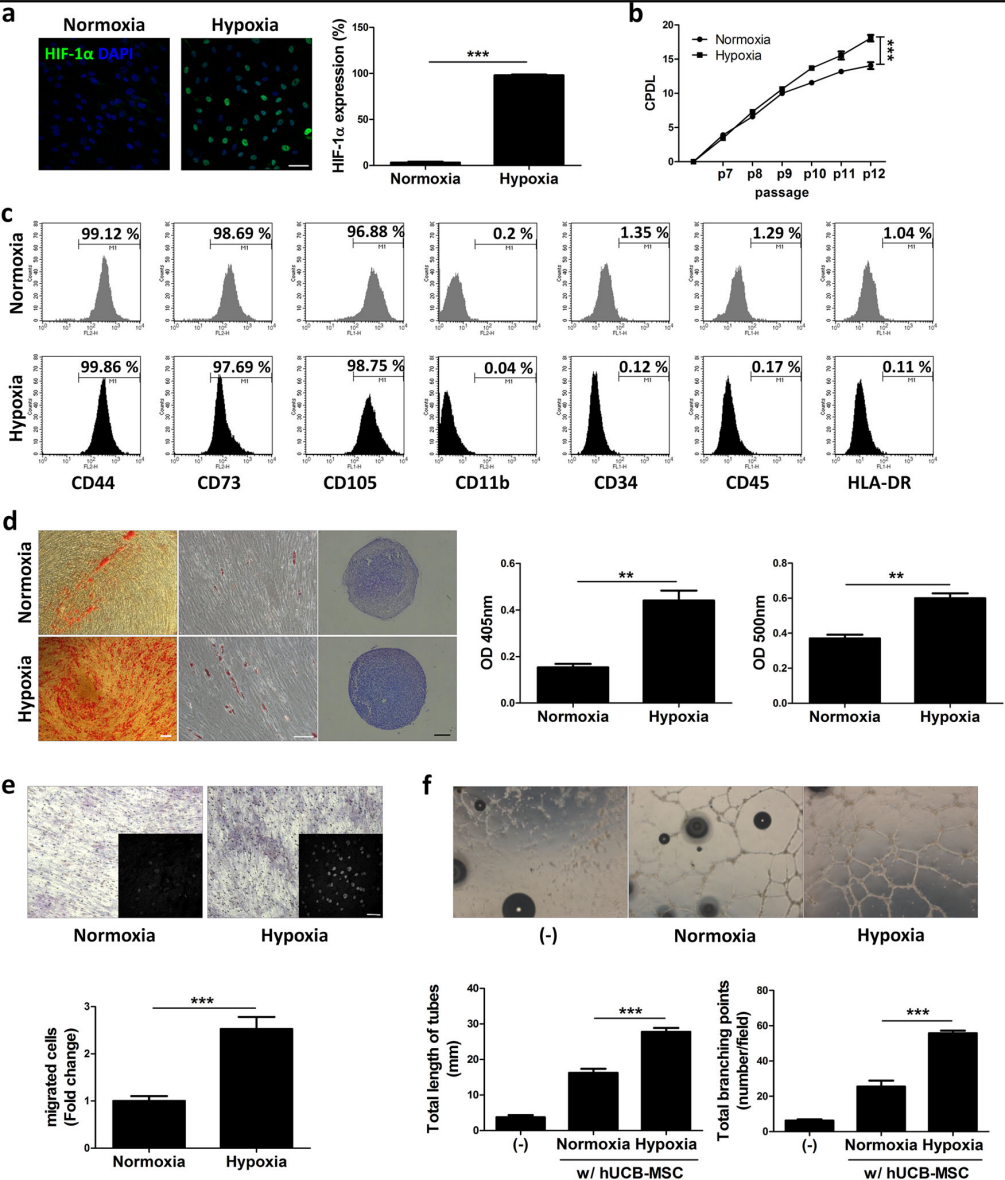
Under hypoxic conditions, hUCB-MSC maintain stem cell characteristics and increase angiogenesis activity. **a** Immunostaining of Hif-1α was performed in hUCB-MSCs in normoxia and hypoxia. Graph indicates the quantification of Hif-1α expression. **b** Cumulative population doubling levels were measured to indicate the proliferation of hUCB-MSC under normoxic and hypoxic conditions. **c** Using flow cytometry, phenotype determination of hUCB-MSC under normoxia and hypoxia were analyzed. **d** Representative images of hUCB-MSCs differentiated into osteogenic, adipogenic, and chondrogenic lineages under normoxic and hypoxic conditions. Osteogenic differentiation is determined by optical density at 405 nm and Alizarin Red S, and adipogenic differentiation was determined by optical density at 500 nm and Oil Red O. **e** Effect of hUCB-MSCs cultured under normoxia and hypoxia on HUVEC migration in a co-cultured transwell system. Migrated HUVECs were stained with hematoxylin and DAPI. Graphs indicate counted migrated HUVECs. **f** Capillary-like tube formation assay of HUVECs co-cultured with hUCB-MSCs cultured under normoxic and hypoxic conditions. Tube formation assay analysis was carried counting total length and branching points of tubes. Scale bars = 50 μm. **P < 0.01; ***P < 0.001. Results are shown as the mean ± SD

To begin, we analyzed the characteristics of hUCB-MSCs when cultured in hypoxic conditions. To confirm the hypoxic conditions, we first examined the enhanced expression of HIF-1α in the nucleus of the hUCB-MSCs when cultured at a low-oxygen level (O_2_ 1%) (Fig. 1a). As reported in our previous study^7^, hUCB-MSCs maintained their proliferating ability when cultured in hypoxic conditions compared to hUCB-MSCs cultured in normoxic conditions (Fig. 1b).

Flow cytometric immunophenotyping of the cells cultured under normoxic and hypoxic conditions did not show any differences in positive markers (CD44, CD73, and CD105) or negative markers (CD11b, CD34, CD45, and HLA-DR) (Fig. 1c). However, when we tested whether hypoxia affected osteogenic, adipogenic, and chondrogenic differentiation, we found different capacities for multi-lineage differentiation (Fig. 1d). Osteogenesis, measured by calcium precipitation using Alizarin Red S, showed elevated stained contents within the cells when cultured under hypoxic conditions. Adipogenesis, measured as the triglyceride content of cells using Oil Red O, was also increased. Finally, chondrogenesis was measured by Toluidine Blue staining of the cartilaginous extracellular matrix, and showed greater density in pellet sections cultured in hypoxic conditions.

The angiogenic properties of hUCB-MSCs were assessed by co-culturing with HUVECs and analyzing tube formation of the HUVECs cultured on Matrigel and the migration ability of the HUVECs. Using a transwell migration assay, we were able to test the HUVEC migration when co-cultured with hUCB-MSCs. The membranes stained with hematoxylin showed more purple area on the membrane co-cultured with hypoxic hUCB-MSCs compared to normoxic hUCB-MSCs (Fig. 1e). To analyze the number of migrated cells, we stained the membrane with DAPI and counted the migrated HUVECs. HUVECs co-cultured with hypoxic hUCB-MSCs showed an elevated number of migrated cells. After 24 h of incubation under hypoxic conditions, the hypoxic hUCB-MSCs were co-cultured with HUVECs on Matrigel in a transwell system. While HUVEC-cultured samples showed less tube formation, hypoxic hUCB-MSCs showed rapid HUVEC tube formation. When compared with hUCB-MSCs cultured in normoxic conditions, hypoxic hUCB-MSCs show increased formation of tubes and branches in HUVECs (Fig. 1f).

### Hypoxic-preconditioned hUCB-MSCs alleviate induced hindlimb ischemia independently of exposure time

Although, it is well known that hypoxic conditioning intensifies the characteristics of MSCs and increases the migration and vascular-like structure-forming capacity of HUVECs^17–20^, as our previous results showed, the optimal culture conditions, including the exposure time, remain unestablished and are widely debated. Therefore, we next investigated whether the exposure time exerted any influence on the augmentation of therapeutic efficacy of hUCB-MSCs in the induced animal model of ischemia.

**Fig. 2:**
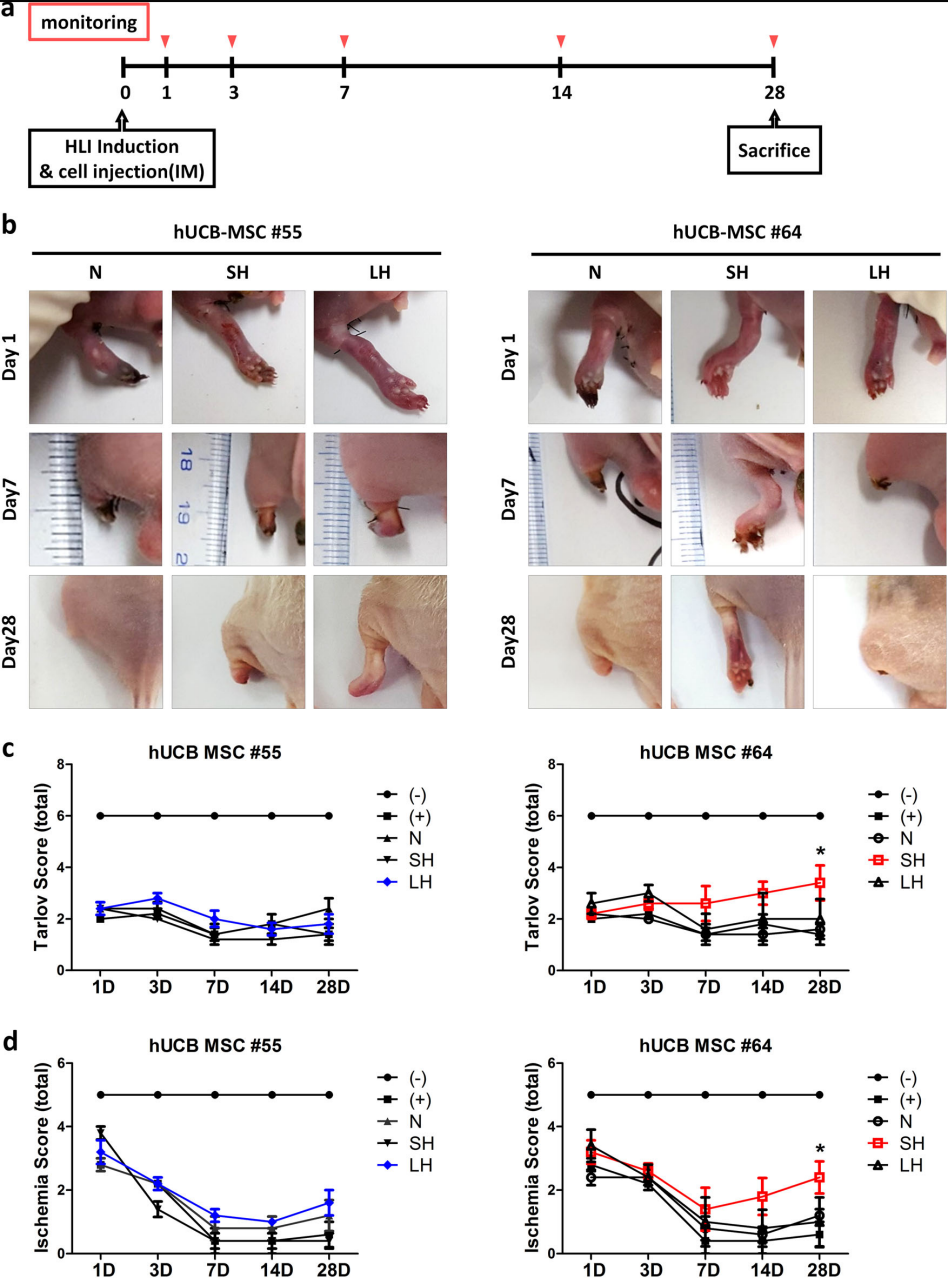
Therapeutic effect of normoxic- and hypoxic-preconditioned hUCB-MSCs in a hindlimb ischemia animal model. Hindlimb ischemia was induced by ligations on the femoral artery and collateral vein. On Day 0, the hUCB-MSCs derived from different donors were injected after normoxic, short term and long term of hypoxic conditioning. **a** Scheme of HLI induction and cell treatment. **b** The HLI-induced mice were monitored on Days 1, 3, 7, 14, and 28, and photographic images of ischemic lesions were collected for gross examination. Functional recovery of cell-injected HLI mice was quantified by the (**c**) Tarlov and (**d**) Ischemia score systems. The results separately show the data from cell-injected groups for better legibility. In vivo experiments conducted under the same experimental conditions and whole-data of photographic images and functional quantifications, including negative and positive control groups are shown in Supplementary Figure S2. Five mice/group were used. *P < 0.05. Results are shown as the mean ± SD

To assess the therapeutic effects, hUCB-MSCs cultured under normoxia or for different hypoxia exposure times (short term (SH): 24 h; long term (LH): 2 weeks) were injected intramuscularly on Day 0 right after the induction surgery (Fig. 2a). Two cell lines derived from different donors were infused to address individual variation. The distribution of injected cells was compared on Day 1. As results, approximately twofolds of SH #64 cells survived and the cells were traced in the injection site (Supplementary Figure 1a and b). After 7 days of hUCB-MSCs administration, the injected cells were not detected in all organs including muscle (Supplementary Figure 1c).

More interestingly, the protective effect represented as foot salvage^21^ was observed only in short-term exposed hUCB-MSCs #64 treated group of mice. There were no significant changes when the other groups of cells were used (Fig. 2b, Supplementary Figure 2a and d). To address functional recovery, the mice were scored using the Tarlov system^14^ at indicated time. In the case of cell line #64, the mice injected with stem cells with short-term exposure to hypoxia showed more improved movement at Day 14 and Day 28 compared to the mice treated in normoxic conditions or long-term exposure to hypoxic cells (Fig. 2c, Supplementary Figure 2b). Similar to the Tarlov test, an application of short-term exposed hUCB-MSCs #64 also led to an alleviation of foot ischemia, which was a typical symptom that accompanies ligations on the femoral artery and collateral vein (Fig. 2d, Supplementary Figure 2c). Even though it did not produce any significant change in the functional movement test, treatment of long-term hypoxic-preconditioned hUCB-MSC #55 showed little effect on tissue recovery of the hindlimb. Taken together, our findings suggest that hypoxic preconditioning cannot be employed as an enhancement strategy for some hUCB-MSCs, and the improvement in therapeutic efficacy is not necessarily related to exposure time.

### Administration of short-term and long-term exposed hUCB-MSCs suppresses infiltration of immune cells and tissue fibrosis

**Fig. 3:**
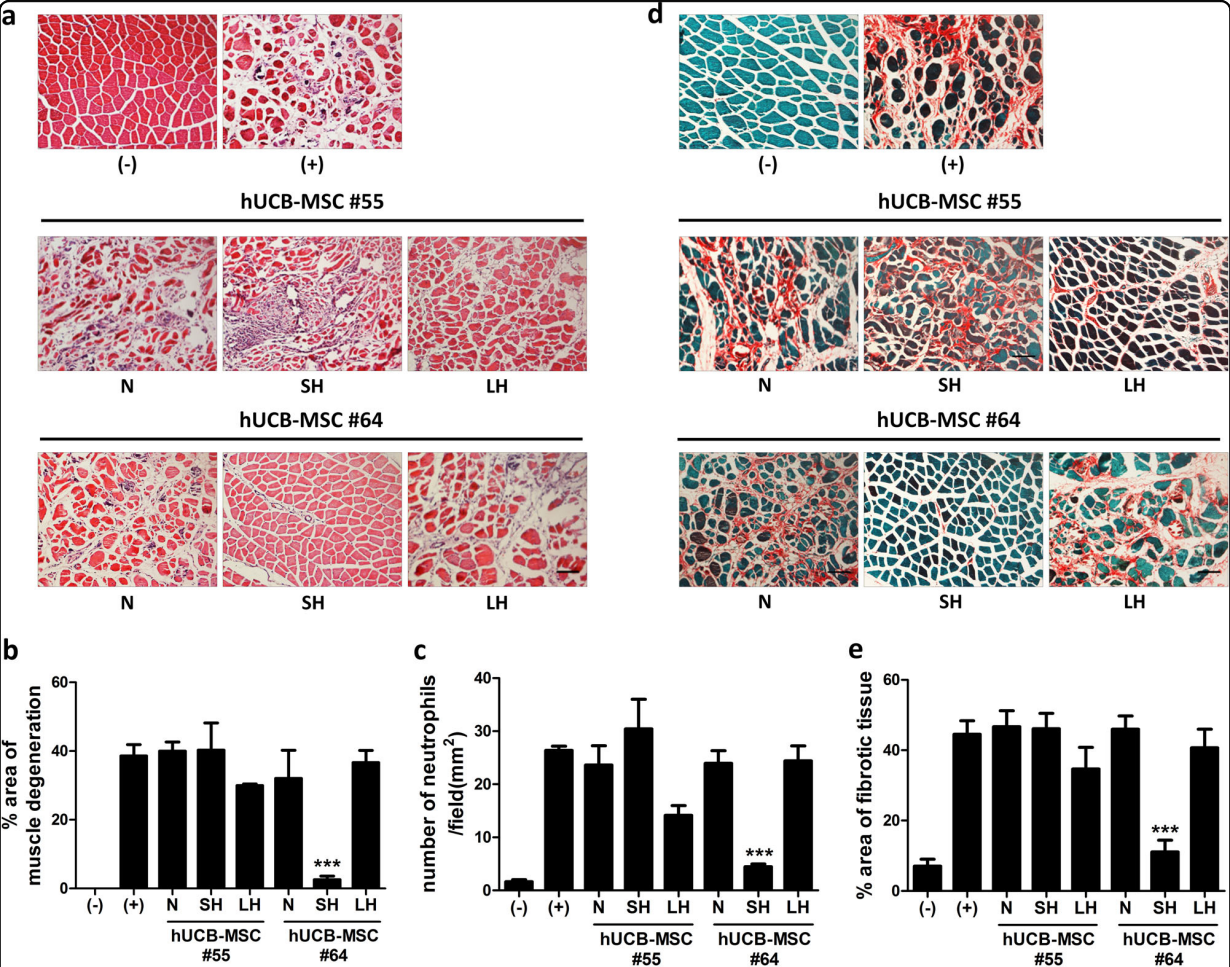
Histopathological assessment of ischemic lesions after normoxic and hypoxic-preconditioned hUCB-MSCs administration. On Day 28, mice were killed for histopathological evaluation. **a** Paraffin-embedded sections of muscle samples from HLI mice were stained with hematoxylin and eosin, and **b** muscle degeneration and **c** the number of infiltrated neutrophils were measured, bar = 200 μm. **d** The sections were stained with picrosirius red, and **e** muscle necrosis were measured, Scale bar = 200 μm. Four to five mice/group were used. ***P < 0.001. Results are shown as the mean ± SD

To confirm the previous results of gross examination and functional tests, we sought to determine the therapeutic potential of hypoxia preconditioned cells by histopathological assessment. The induced ischemia model mice were killed at day 28, and the hind limbs of the mice were collected for H&E and picrosirius red staining.^22,23^ Histopathological examination using H&E staining revealed that tissue degeneration, including loss-of-muscle fibers and muscle atrophy, was dramatically attenuated by intramuscular deliver of SH #64, and the attenuation was seen with LH #55 to some degree (Fig. 3a). To quantify the alleviation of muscle degeneration, the ratio of non-muscular area to total area was measured. As the results showed, ~40% of the degenerative region in the positive control group was decreased by 10% after LH #55 treatment, and the ratio in the SH #64 treated group only accounted for 1.57% (Fig. 3b). Immune cell infiltration in the ischemic region was also rescued by application of SH #64 and LH #55 (Fig. 3a, c). Muscle fibrosis caused by ischemic tissue damage was also addressed by picrosirius red staining and quantification of fibrotic area. Similar to muscle degeneration and immune cell infiltration, SH #64 mice displayed almost the same level of fibrosis as normal mice, and LH #55 reduced muscle fibrosis somewhat compared to other groups (Fig. 3d, e). These results indicate that, similar to the gross and functional analyses, short-term hypoxia exposure with hUCB MSCs #64 dramatically suppressed muscle degeneration, local immune cell infiltration and fibrosis caused by induced ischemia, and the decreased, but similar, amelioration tended to appear with treatment with LH #55.

### Hindlimb angiogenesis in the PAD model improved after administration of hypoxia-conditioned hUCB-MSCs

**Fig. 4:**
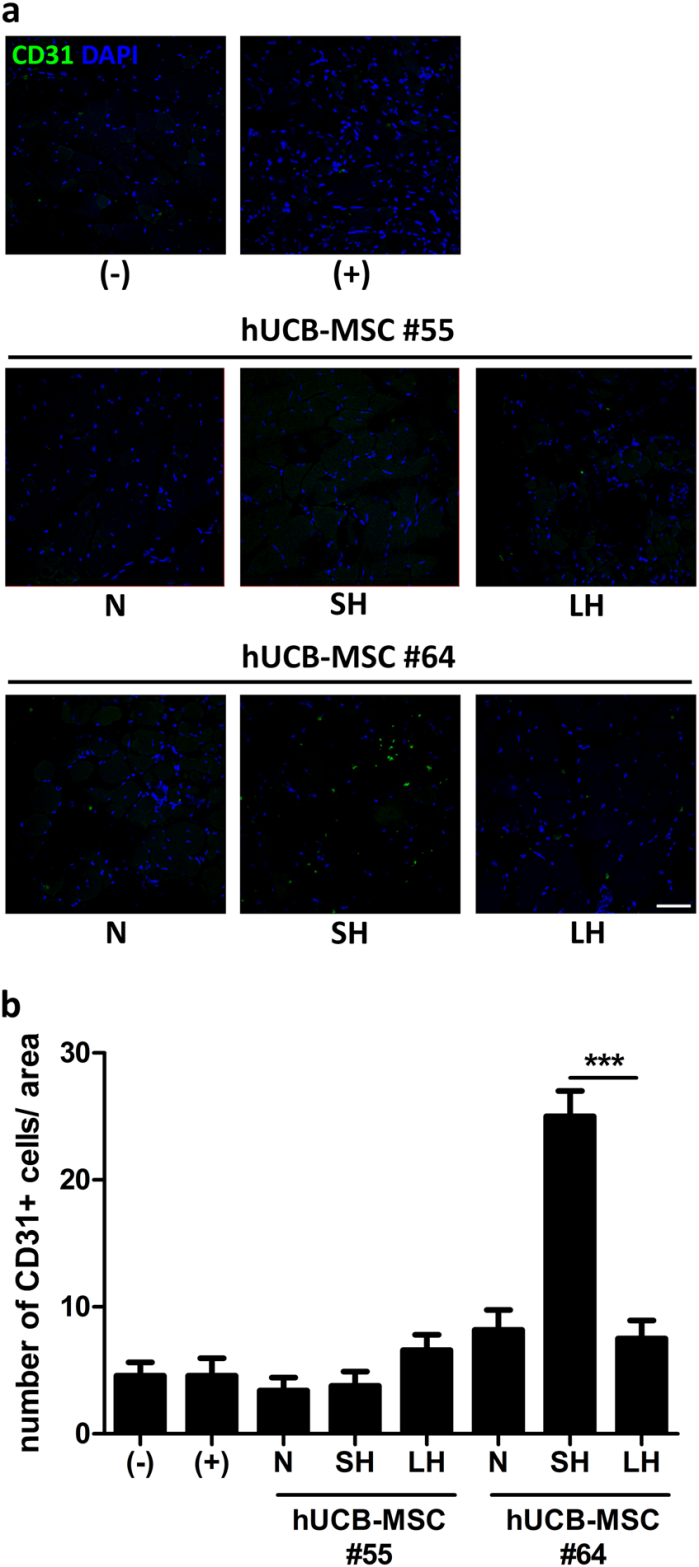
Promoted hindlimb angiogenesis of the ischemic lesions show different effects among culture conditions and donors. **a** Representative image of CD31-positive blood vessels (green) from the adductor muscle section among groups. Scale bar = 50 μm. **b** Quantification of CD31-positive capillaries among groups. ***P < 0.001. Results are shown as the mean ± SD

To estimate hindlimb functional recovery, the number of microvessels was determined by analysis of the endothelial cell marker, CD31. Mice from each group were killed on day 28 for histological staining with CD31 (Fig. 4). As expected, there was no indication of CD31-positive vessels in mice treated with hUCB-MSCs #55. Only a small amount of single-positive vessels was detected in LH#55. However, in the group treated with hUCB-MSCs #64, there was significantly improved density of CD31-positive vessels in SH #64 compared with that of the hUCB-MSCs #55 group. These data show analogous results in Figs. 2 and 3, indicating muscle degeneration and immune cell infiltration are correlated with capillary counts around the muscle area.

### hUCB-MSCs promote endothelial tube formation and migration in a donor-dependent manner

**Fig. 5:**
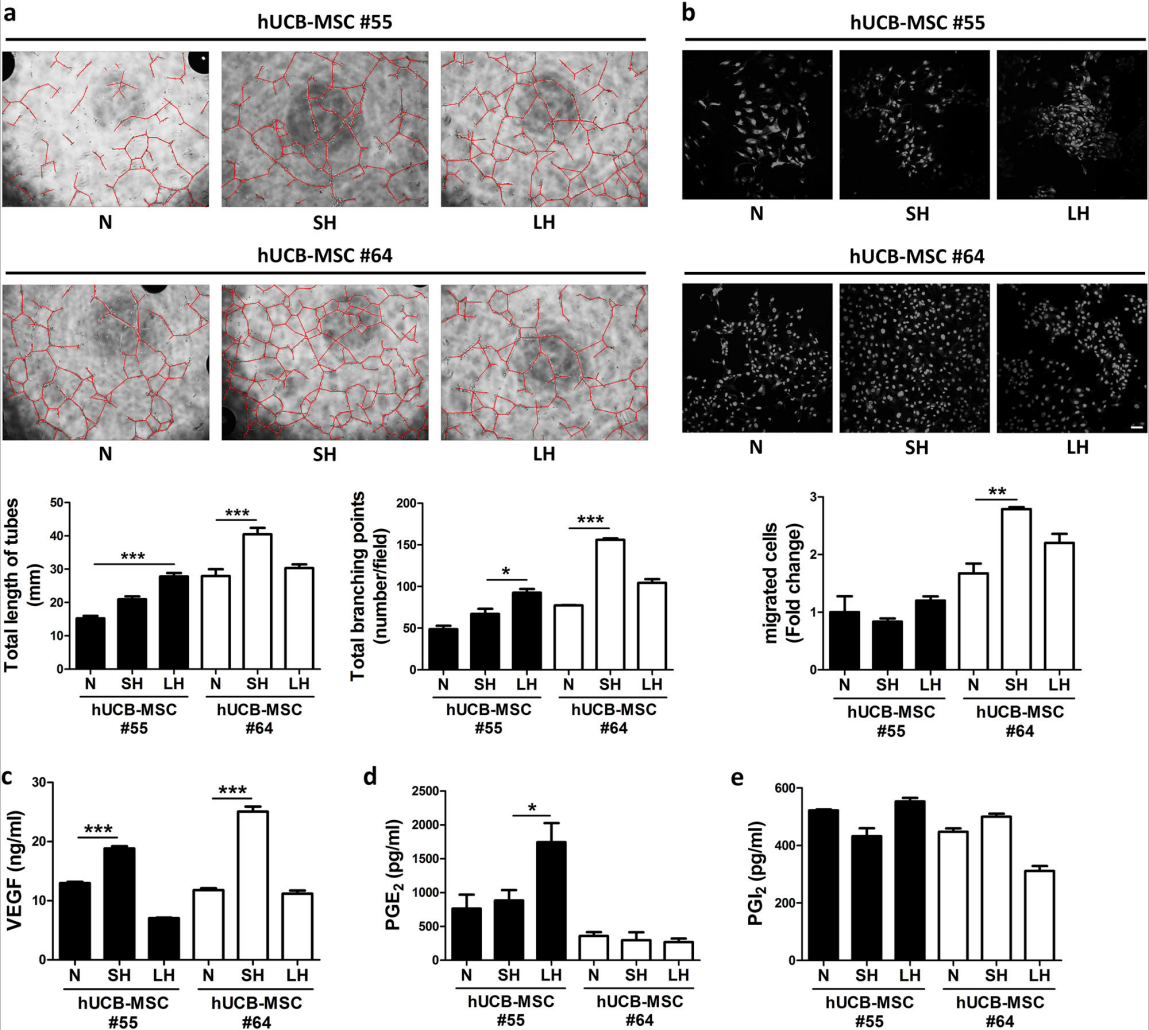
In vitro angiogenesis capacity is altered between culture condition and donor. **a** Capillary-like tube formation assay of HUVECs co-cultured with hUCB-MSC #55 or #64. Tube formation was quantified by total tube formation length and branch junction. Original version of images are shown in Supplementary Figure S3. **b** Migration assay using HUVECs co-cultured with hUCB-MSCs. Graphs show migrated HUVECs by total cell counting. **c**–**e** VEGF, PGE_2_, and PGI_2_ concentrations were measured from the cultured media of hUCB-MSCs by ELISA. Scale bars = 50 μm. *P < 0.05; **P < 0.01; ***P < 0.001. Results are shown as the mean ± SD

To determine whether donor variation affects the angiogenic properties of hUCB-MSCs, we investigated how it affected the angiogenesis-promoting function of HUVEC tube formation by culturing HUVECs on Matrigel in the presence of hUCB-MSCs in a transwell co-culture system. After a 24-h incubation, tube formation was imaged by phase contrast microscopy (Fig. 5a, Supplementary Figure 3). This assay revealed that hUCB-MSC #64 enhanced tube formation of HUVECs compared to hUCB-MSC #55. SH#64 was most effective in tube formation, and both SH#55 and LH#55 showed less angiogenesis in tube total length and branch formation.

HUVEC migration was observed by performing a transwell migration assay (Fig. 5b). hUCB-MSC #55 promoted only a small amount of HUVEC migration. Hypoxic conditioning on #55 (SH#55 and LH#55) produced no significant change in HUVEC migration. Compared with the hUCB-MSC #55 group, the hUCB-MSC #64 group had substantially greater changes in HUVEC migration. Along with HUVEC tube formation, SH#64 showed a compelling increase in HUVEC migration, meaning hypoxia-conditioned hUCB-MSCs improved the angiogenic promotion properties. However, according to HUVEC tube formation and migration assays, hypoxia-conditioned hUCB-MSCs could not overcome donor variation effects on the angiogenic properties.

Given that VEGF represents a standard therapeutic target in angiogenesis and related diseases, we analyzed the secreted VEGF protein in hUCB-MSC #55 and hUCB-MSC #64 with their hypoxia-conditioned groups using ELISA (Fig. 5c). Both hUCB-MSCs showed increased VEGF secretion in SH groups and less secretion in LH groups. However, there were no significant differences between donors, and there were relatively similar secretion patterns among #55 and #64. We additionally analyzed different angiogenic factors, such as PGE_2_ and PGI_2_, which are prostanoids possessing angiogenic activity (Fig. 5d, e)^24^. In the case of PGE_2_, elevated secretion in hUCB-MSC #55 was found only in the long-term hypoxic culture, whereas no significant changes in hUCB-MSC #64 were observed. Taken together, there were no representative patterns of well-known secreted angiogenic factors explaining the difference between donors in the angiogenic properties shown in in vivo and in vitro assays.

### Comprehensive analysis of hypoxia preconditioned hUCB-MSCs using global gene expression profiling identified an angiogenic gene expression signature that was donor-dependent

To characterize the hypoxia preconditioned hUCB-MSC phenotype, we performed a genome-wide NGS analysis using total RNA transcriptomes from all six samples, namely, N#55, SH#55, LH#55, N#64, SH#64, and LH#64. The global genome heatmap with hierarchical cluster analysis showed a significant difference between N#64 and SH#64 and a high similarity between LH#64 and LH#55 and between N#55 and SH#55 (Supplementary Figure 4a). Analogous to these global gene expression patterns, another hierarchical clustering consisted of genes in HIF-1 and VEGF signaling pathways that showed a significant difference between N#64 and SH#64 and a high similarity between LH#64 and LH#55 and between N#55 and SH#55 (Supplementary Figure 4b). It is of note that many representative hypoxia-induced genes were strongly upregulated in the hUCB-MSC line #64, whereas significant molecular changes did not occur in the hUCB-MSC line #55.

We further compared the global gene expression patterns between N#64 and SH#64 by GO function enrichment analysis^25^. Genes that showed a more than twofold alteration were included in the analysis, and their relevant GO categories are illustrated in Supplementary Figures 5a and b. Not surprisingly, there were no significant enrichments in biological processes. However, the expression of genes upregulated in SH#64 were related to blood circulation, macrophage activation, anatomical structure morphogenesis and the transmembrane receptor protein tyrosine kinase signaling pathway (Supplementary Figure 4c, red). On the other hand, the expression of genes downregulated in SH#64 was related to rRNA and rRNA metabolic processes, protein targeting and nuclear transport (Supplementary Figure 4c, blue). By comparing N#55 and LH#55, such upregulation of genes related to blood circulation was not detected in the hypoxia preconditioned hUCB-MSC #55 (Supplementary Figure 4d). Interestingly, two hUCB-MSC lines differed in intrinsic gene expression patterns identified by analysis of genes related to biological processes (Supplementary Figure 4e). The hUCB-MSC #64 in the normoxic culture condition showed higher enrichment of genes related to blood coagulation and angiogenesis than N#55.

To further detect the effect of donor-dependent variations on angiogenesis in our in vivo induction protocol, we quantitatively analyzed the gene expression in all six samples, N#55, SH#55, LH#55, N#64, SH#64, and LH#64, with NGS analysis. We selected genes showing a higher intrinsic expression in N#64 compared to N#55. Among these genes, the genes that showed the highest fold enrichment by GO function enrichment analysis above (Supplementary Figure 4e) were further included in the analysis with the six individual samples (Supplementary Figure 5c-f). Such genes were categorized as a DNA binding protein, particularly a homeobox transcription factor. All 18 homeobox transcription factors were expressed at a higher gene expression level in all #64 samples than #55 samples. Nevertheless, they did not show consistent changes in gene expression patterns after hypoxic preconditioning. Therefore, we determined that DNA binding proteins, which showed the highest fold enrichment, produce donor-dependent variations in limb ischemia.

**Fig. 6:**
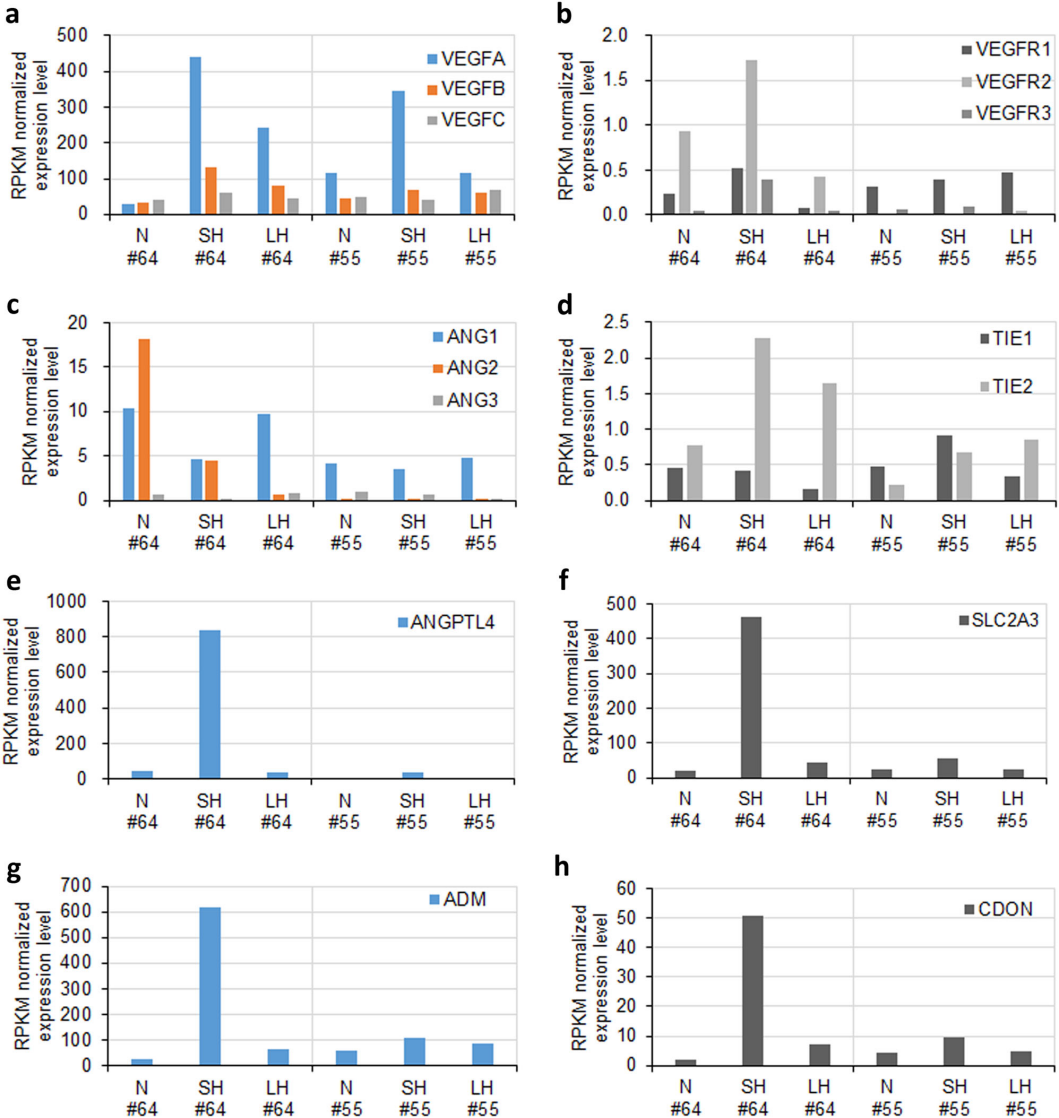
Genome-wide NGS analysis provides a clue to donor-dependent variations in stem cell therapy for limb ischemia. **a**–**h** Expression levels of transcriptomes in six samples N#55, SH#55, LH#55, N#64, SH#64, and LH#64 were analyzed by NGS analysis. The mRNA sequencing analysis was indicated normalized gene expression changes of angiogenic factors (VEGFA, VEGFB, VEGFC, ANG1, ANG2, ANG3 and ANGPTL4; color) and their receptors (VEGFR1, VEGFR2, VEGFR3, TIE1 and TIE2; gray), as well as additional markers (SLC2A3, ADM, and CDON) in both hUCB-MSC lines #64 and #55 as compared to the hypoxic-preconditioned SH- or LH#64 and SH- or LH#55, respectively

Next, we quantitatively analyzed the expression of angiogenic factors and their receptors. First, we investigated angiogenic growth factors (VEGFA, VEGFB, and VEGFC) and their receptors (VEGFR1, VEGFR2, and VEGFR3). We found that the hypoxia elevated VEGFA gene expression in both SH#64 and SH#55 but did not change *VEGFB* and *VEGFC* gene expression in either cell line (Fig. 6a). Interestingly, the expression of a major VEGF receptor, VEGFR2, was inherited only in #64 and upregulated by hypoxia (Fig. 6b). Second, we investigated angiopoietins (ANG1, ANG2, and ANG3) and their receptors (TIE1 and TIE2). We found that ANG2 gene expression was inherited only in #64 (Fig. 6c). The hypoxic preconditioning could elevate the gene expression of its receptor TIE2; whereas, it did not regulate gene expression of any angiopoietins in #55 (Fig. 6d). Third, we investigated peroxisome proliferator-activated receptors (PPAR-α, PPAR-Δ, and PPAR-γ) and found that the *PPAR-γ* gene expression was inherited at a higher level in N#64 (Supplementary Figure 5g). However, these changes in gene expression patterns in the six samples showed barely at a basal level.

To further explore our hypothesis that each donor of hUCB-MSCs had different genetic backgrounds and showed donor-dependent effects on stem cell therapy outcomes, we attempted to identify genetic markers for hypoxia-induced pro-angiogenic effects. Of entire gene expression data from the NGS analysis, we selected 10 genes expressing at a more than fivefold higher level in the hypoxia condition SH#64, compared to any other samples N#55, SH#55, LH#55, and LH#64 (Supplementary Figure 6a-j). From the list of genes, we sorted out four genes based on previous studies on the relation in angiogenesis and the sensitivity on hypoxic condition, angiopoietin-like 4 (ANGPTL4), adrenomedullin (ADM), and glucose transporter 3 (GLUT3, also known as SLC2A3), as well as a cell adhesion molecule-related factor (CDON). These four markers showed that its gene expressions were dramatically upregulated only in #64 upon hypoxic conditioning, whereas such upregulations have not been observed in #55 hUCB-MSC line (Fig. 6e–h, Supplementary Table 3). This change of gene expression pattern demonstrated that #64 was susceptible to the hypoxic preconditioning.

### The in vitro angiogenesis capacity of hUCB-MSCs is not affected by donor gender or health condition but by gene expression patterns

**Fig. 7:**
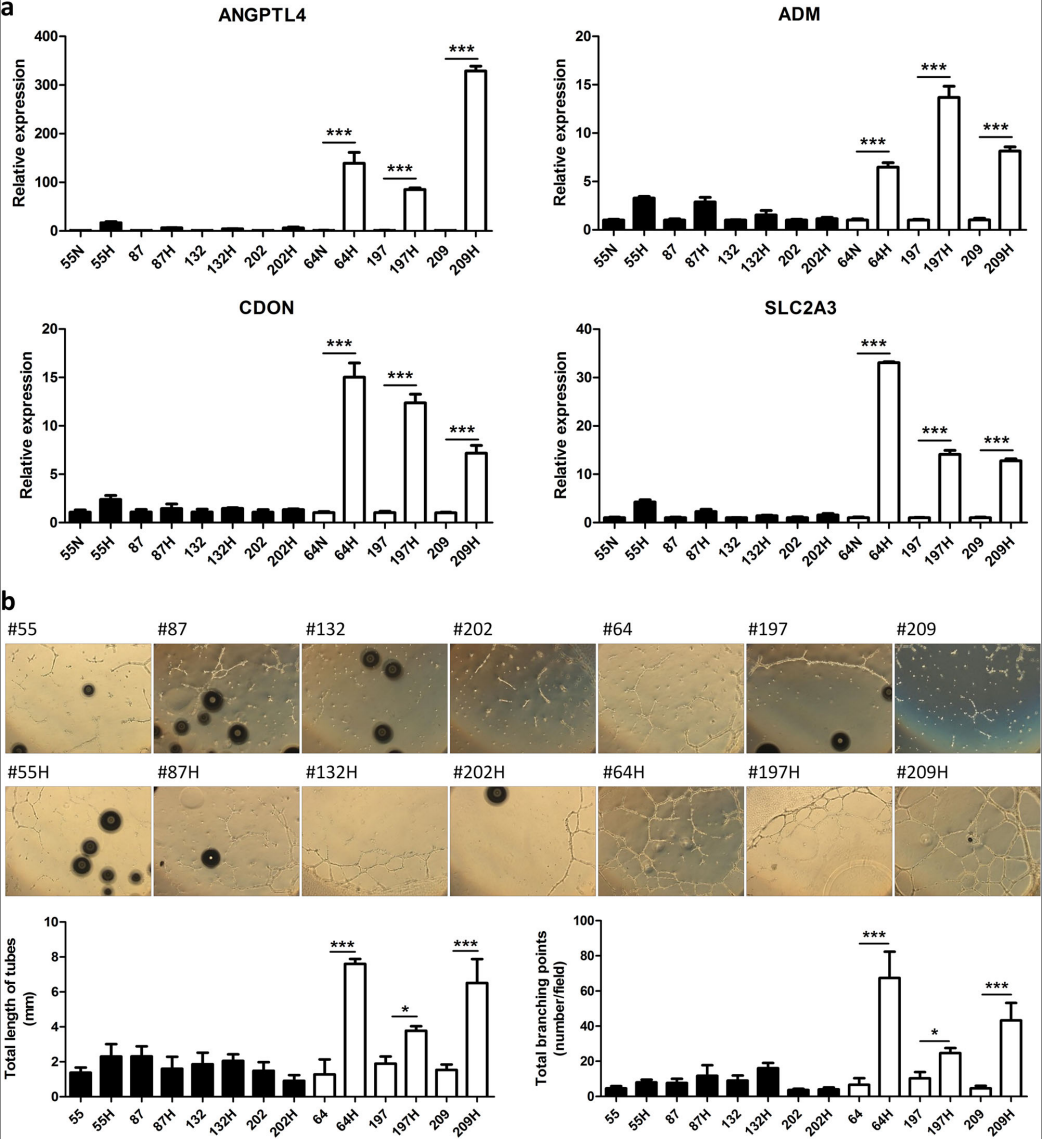
Validation of selected genes expression by qPCR in low- and high-angiogenesis capacity hUCB-MSCs. **a** Validation of gene expression of genes selected form NGS (ANGPTL4, ADM, CDON, and SLC2A3) was analyzed by qPCR and show significant different expression pattern between low-angiogenesis capacity hUCB-MSCs and high-angiogenesis capacity hUCB-MSCs. **b** Capillary-like tube formation assay of HUVECs co-cultured with hUCB-MSCs #55, #64, #87, #132, #197, #202, #209 was performed and revealed two type of groups, low-angiogenesis capacity (#55, #87, #132, #202) and high-angiogenesis capacity (#64, #197, #209). The angiogenesis capacity of hUCB-MSCs was determined by quantification of tube formation length and branch junction. Scale bars = 100 μm. *P < 0.05, ***P < 0.001. Results are shown as the mean ± SD

To determine whether the expression levels of selected four factors (ANGPTL4, ADM, CDON, and GLUT3) are changed in a donor-dependent manner, hUCB-MSC #55, #64, #87, #132, #197, #202, and #209 were cultured in hypoxic condition and quantitative PCR analysis was conducted. Gender and health condition of the donors are listed in Table 1. All hUCB-MSCs from different donors expressed ANGPTL4, ADM, CDON, GLUT3 transcripts. When cultured in normoxia condition, hUCB-MSCs tend to express a standardized levels of ANGPTL4, ADM, CDON, GLUT3 transcripts. However, after treatment of hypoxic condition, gene expression patterns were divided into two groups. hUCB-MSCs #55, #87, #132, #202 show less increase of gene expression levels of ANGPTL4, ADM, CDON, GLUT3, while hUCB-MSCs #64, #197, #209 show significant increase of gene expression levels of those four factors after hypoxic culture (Fig. 7a). To validate whether the four factors were crucial for selection of hUCB-MSCs for therapeutic usage, we conducted a capillary-like tube formation assay of HUVECs co-cultured with hUCB-MSCs (#55, #64, #87, #132, #197, #202, #209). Quantification of HUVEC tube formation led to categorize the hUCB-MSCs into two types of group, low-angiogenesis capacity (#55, #87, #132, #202) and high-angiogenesis capacity (#64, #197, #209) (Fig. 7b). hUCB-MSCs with high-angiogenic capacity were determined by significant increase in tube formation after hypoxic conditioned culture. Whereas hUCB-MSCs with low-angiogenic capacity did not cause any significant increase in total length or branching formation of HUVEC. Taken together, these four genetic markers away from secreted protein markers, such as VEGF, PGE_2_, and PGI_2_, could properly indicate which cell lines are susceptible to the hypoxic preconditioning.

**Table 1:** General information of hUCB-MSCs

No.	Cell line	Sex	Illness
**1**	#55	Female	No disease-related phenotypes
**2**	#64	Male	No disease-related phenotypes
**3**	#87	Male	No disease-related phenotypes
**4**	#132	Male	No disease-related phenotypes
**5**	#197	Male	No disease-related phenotypes
**6**	#202	Male	No disease-related phenotypes
**7**	#209	Male	No disease-related phenotypes

## Discussion

Although a number of studies has reported that hypoxic preconditioning increases therapeutic potential and the cellular functions of mesenchymal stem cells, including differentiation, motility, and vascular forming capacity, these studies recruited their own experimental conditions, such as the hypoxia concentrations and exposure times^18,19,22,26^.

To employ hypoxic preconditioning as an enhancement strategy for therapeutic application of the cells beyond academic elucidation of cellular mechanisms, we thought that unified optimal conditions needed to be established and that the therapeutic efficiency of the conditions must be scientifically verified. To do so, we administered hUCB-MSCs with different hypoxia exposure times to induced hindlimb ischemia with two cell lines from different donors. As many studies reported previously, the application of hypoxia pretreated cells could improve pathological signs compared to normoxia cultured cells^27–30^. Hung et al.^31^ reported that human MSCs increased their therapeutic potential by upregulating the expression level of chemokine receptors and their engraftment in vivo when exposed short-term to hypoxia. In contrast, Saller et al. demonstrated that similar effects were attained by 2 weeks of hypoxic culture, and increased stemness and migration of human MSCs were associated with regulation of integrin expression^32^. However, we did not find exposure time-dependent effects on the alleviation of ischemia in this animal model. In the case of hUCB-MSC #55, long-term hypoxic exposure exerted a remedial value on ischemia-mediated tissue damage. On the other hand, hUCB-MSC #64 showed improved tissue protection when it was exposed to short-term hypoxia. We achieved similar results from histopathological assessment, which indicated muscle regeneration and local inflammation. However, interestingly, the restoration of motor function only appeared in the group injected with SH #64 and not LH #55. Different from a number of previous studies, we found that tissue recovery around ischemic lesions was not always followed with restoration of functional movement from these results. In addition, the factors or mechanisms that caused this discrepancy need to be addressed.

More importantly, the recovery of the lesion area was followed with a high density of CD31-positive capillaries. Induced angiogenesis capacity was shown only in the group injected with SH #64, and this result from the in vivo experiment further confirmed in in vitro HUVEC tube formation and migration assays. Matrix tube formation and migration is a common assay the analyze angiogenic capabilities. As expected, SH #64 showed the most elevated HUVEC network formation and migration results. These data are consistent with in vivo data, meaning hypoxic preconditioning for a long or short period could not overcome any donor-dependent effects. We suggest that the angiogenic properties of human MSCs were different between donors, and these properties were unable to overcome hypoxic preconditioning. Although most of the studies reported that therapeutic effects of hypoxia preconditioned stem cells were mediated by an increase in secretory factors such as VEGF, PGE_2_, and PGI_2_^33–35^, we showed here that actual enhancement of therapeutic outcomes did not correlate with those factors. The secretion of VEGF, which is well-known to be a prominent factor that responds to hypoxia and HIF-1alpha, was increased with short exposure in both 2 cell lines.

In case of PGE_2_, the LH #55 alone responded to hypoxic stimulation, and the changes in PGI_2_ reflected the therapeutic tendency somewhat but were not significant. Based on our data, hypoxic preconditioning mediated in vitro and in vivo improvement but was not fully explained by those factors alone. Thus, additional factors or more specific mechanisms are needed to demonstrate the actual therapeutic effect beyond a preclinical study. In this study, we demonstrated that different populations of hUCB-MSCs can respond differently to angiogeneic stimulation, and this response was consistent throughout in vitro and in vivo assays, meaning that in vitro potency assays in angiogenesis are reliable for predicting cell function *in vivo*. The present study focused on donor-to-donor heterogeneity of hUCB-MSCs. Functional discordance of human MSCs among cell populations from different donors have been reported previously^36^. Although functional differences have been reported, the results were not associated with the cell source or different patterns of cell surface marker expression. We analyzed the angiogenetic portion of hUCB-MSCs and discovered that well-established angiogenic factors offer limited explanation of the intra-population heterogeneity of hUCB-MSCs. Because the use of human MSCs in clinical medicine continues to grow rapidly, it is important to understand the donor-dependent characteristics of hUCB-MSCs.

To address the issue of donor-dependent variations in stem cell therapy, we explored further different genetic backgrounds between both hUCB-MSC lines #64 and #55. By selecting of representative markers for hypoxia-induced pro-angiogenic effect, we figured out four genes, including two well-known hypoxia-induced angiogenic factors ANGPTL4 and ADM and GLUT3, as well as a cell surface receptor CDON. ANGPTL4 protein belongs to a super family of secreted proteins structurally related to factors modulating angiogenesis^37^. ADM activates the PI3K/Akt-dependent pathway in vascular endothelial cells to regulate multiple critical steps in angiogenesis^38^. HUVEC treated with GLUT3 siRNA revealed 27% reduction in tube formation and deletion of CDON results a corresponding failure to promote angiogenesis^39,40^. All four genes did not show biased expression patterns between N#64 and N#55 hUCB-MSC lines. However, these four genes were expressed significantly more in SH#64 than any others. Based on these, we investigated whether the four marker genes truly correlated with pro-angiogenic property of hUCB-MSCs. By the analysis using seven donors (1 female and 6 males in a healthy condition), we propose that pro-angiogenic property of hUCB-MSCs can be determined by specific gene expression profile.

The present study revealed that hUCB-MSCs have donor-dependent individual differences and do not respond to hypoxic environments identically. As described above, donor-dependent variations cannot be clearly elucidated by well-known, secreted angiogenic factors. Thus, optimization and selection based on comprehensive genome-wide gene expression analysis is needed for actual therapeutic application. Here we showed that hypoxic-preconditioned hUCB-MSCs possessed distinctive expression patterns of specific genes (ANGPTL4, ADM, CDON, and GLUT3) with a donor-to-donor variability. Given that the expression pattern represents the pro-angiogenic property of hUCB-MSCs, we suggest using selected genetic factors as general indicators to guarantee successful stem cell therapy.

## Electronic supplementary material

Supplementary Information
