# Supplementary material for: Donor-dependent variation of human umbilical cord blood mesenchymal stem cells in response to hypoxic preconditioning and amelioration of limb ischemia
Source: Exp Mol Med. 2018 Apr 20;50(4):35. doi: 10.1038/s12276-017-0014-9 (PMC5938050; doi:10.1038/s12276-017-0014-9)
Supplement: Supplementary file 1 — Supplementary Information [file 12276_2017_14_MOESM1_ESM.pdf]

Donor-dependent Variation of Human Umbilical Cord Blood Mesenchymal Stem Cells in Response to Hypoxic Preconditioning and Amelioration of Limb Ischemia

Authors and affiliations

Insung Kang[1,2*], Byung-Chul Lee[1,2*], Soon Won Choi[1,2*], Jin Young Lee[1,2], Jae-Jun Kim[1,2], Bo-Eun Kim[1,2], Da-Hyun Kim[1,2], Seung Eun Lee[1,2], Nari Shin[1,2], Yoojin Seo[1,3,4], Hyung-Sik Kim[1,3,4], Dong-Ik Kim[5] and Kyung-Sun Kang[1,2†]

[1]Adult Stem Cell Research Center, College of Veterinary Medicine, Seoul National University, Seoul 08826, South Korea

[2]Research Institute for Veterinary Medicine, College of Veterinary Medicine, Seoul National University, Seoul 08826, South Korea

[3]Pusan National University School of Medicine, Busan 49241, South Korea

[4]Biomedical Research Institute, Pusan National University Hospital, Busan 49241, South Korea

[5]Division of Vascular Surgery, Samsung Medical Center, Sungkyunkwan University School of Medicine, Seoul 06351, South Korea

[*] These authors contributed equally to this work

[†]Correspondence

Kyung-Sun Kang, D.V.M., Ph.D.

Adult Stem Cell Research Center, College of Veterinary Medicine, Seoul National University, 1 Gwanak-ro, Gwanak-gu, Seoul 08826, South Korea

Tel. +82-2-880-1246

E-mail: kangpub@snu.ac.kr

Total number of words: 5,490 words

Total number of figures: 7 (6 additional supplementary figures)

Total number of tables: 1 (3 additional supplementary tables)

Supplementary Table 1. Tarlov Scale

Tarlov Scale	
0	No movement
1	Barely perceptible movement, non-weight bearing
2	Frequent movement, non-weight bearing
3	Supports weight, partial weight bearing
4	Walks with mild deficit
5	Normal but slow walking
6	Full and fast walking

Supplementary Table 2. Ischemia scale

Ischemia Scale	
0	Auto-amputation > half lower limb
1	Gangrenous tissue > half foot
2	Gangrenous tissue < half foot, with lower limb muscle necrosis
3	Gangrenous tissue < half foot, without lower limb muscle necrosis
4	Pale foot or gait abnormalities
5	Normal

Supplementary Table 3. Normalized expression levels of Hypoxia-induced overexpressed genes in SH#64

No.	Gene name	N#64	SH#64	LH#64	N#55	SH#55	LH#55
**1**	ANGPTL4	45.47	836.55	40.75	5.54	34.24	8.67
**2**	ADM	27.73	618.02	61.07	60.31	105.52	85.39
**3**	SLC2A3	20.62	464.49	42.48	23.43	54.54	23.35
**4**	CDON	1.73	50.89	7.26	4.16	9.5	4.59

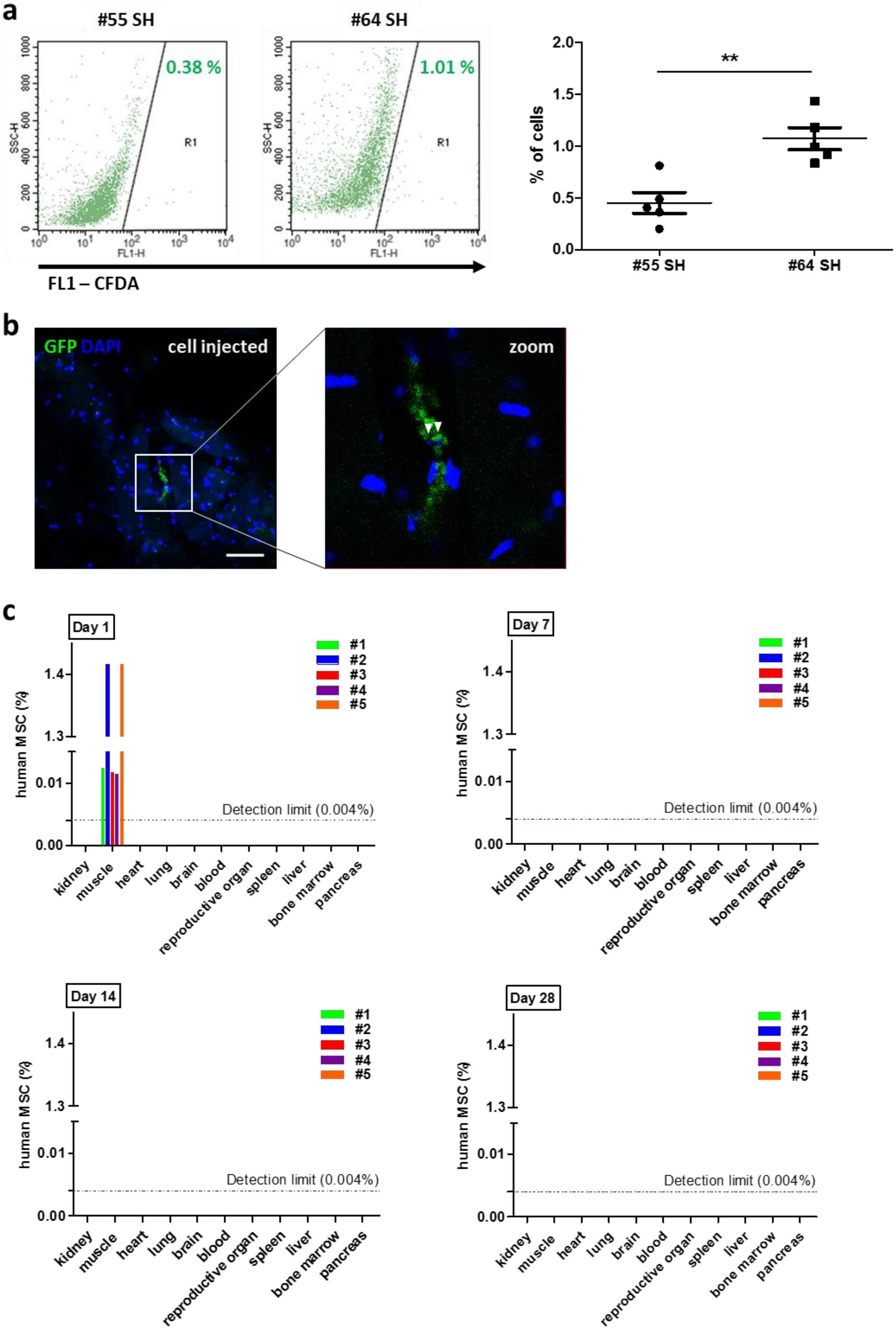

a
#55 SH
#64 SH
0.38 %
1.01 %
R1
SSC-H
FL1-H
FL1 – CFDA
% of cells
**
#55 SH
#64 SH
b
GFP DAPI
cell injected
zoom
c
Day 1
Day 7
Day 14
Day 28
#1
#2
#3
#4
#5
human MSC (%)
Detection limit (0.004%)
kidney
muscle
heart
lung
brain
blood
reproductive organ
spleen
liver
bone marrow
pancreas

Supplementary Figure S1. Distribution of hUCB-MSCs infused to hindlimb ischemia induced mice (a) Distribution of hUCB-MSCs (SH #55 and SH #64) locally injected into mice hindlimb muscle was quantified by flow cytometry. (b) hUCB-MSCs transfected with shRNA for GFP were injected intramuscularly and traced by confocal microscope from muscle sections, cells were indicated as ▼, Bar = 50 μm. (c) Distribution of the delivered hUCB-MSCs in various organ of HLI mice was defined on indicated time point by qRT-PCR using human-specific ALU gene. Results show representative images. Five mice/group were used. ** $P<0.01$. Results are shown as the mean ± SD.

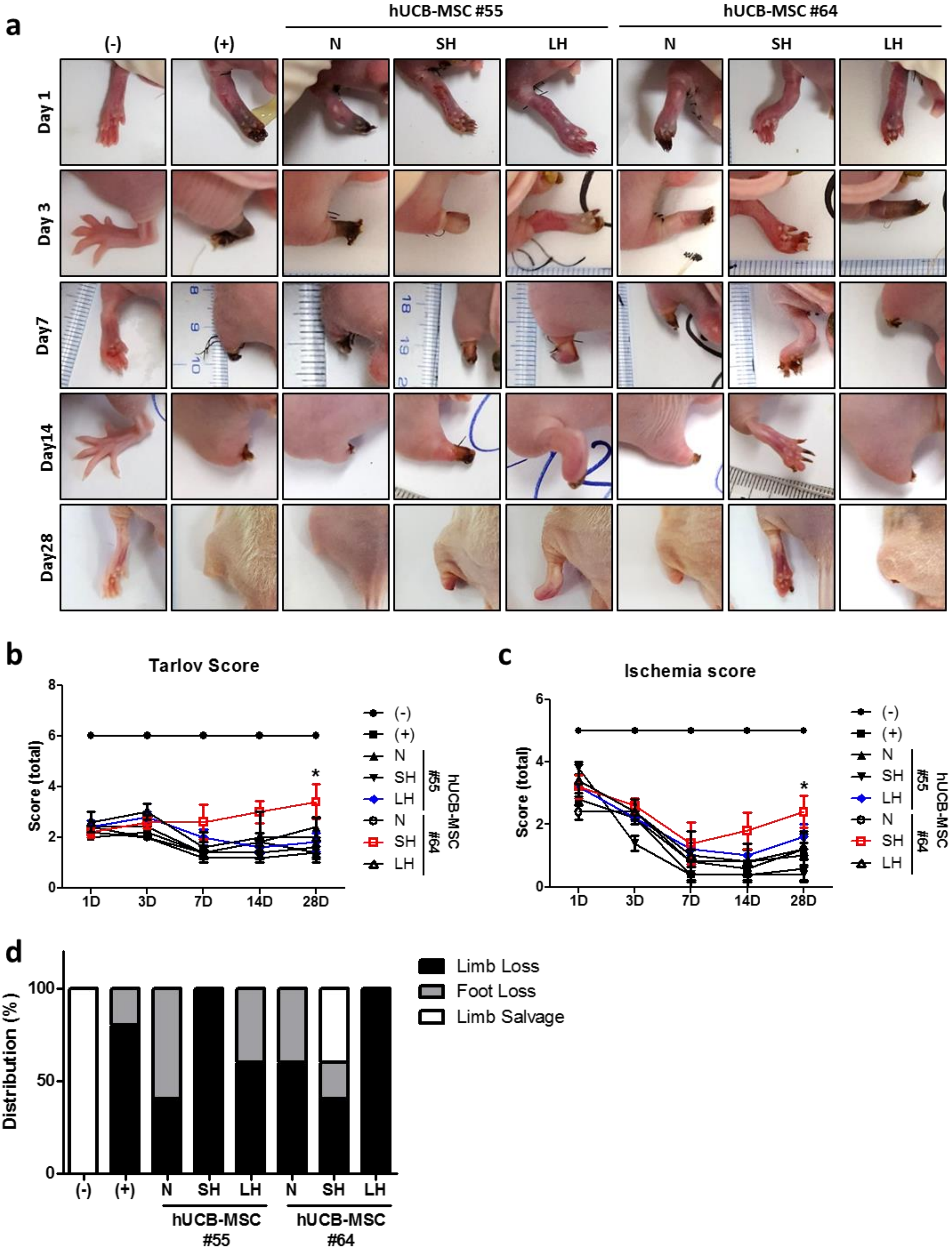

Supplementary Figure S2. Entire data of gross and functional examination from *in vivo* experiments (a-c) Whole data of (a) photographic images, (b) Tarlov score and (c) ischemia score of HLI induce mice. Results show representative images. (d) On Day 28, pathophysiological status of ischemic limbs were evaluated by classifying the limbs into three grades (Limb loss, foot loss and foot salvage). * $P<0.05$. Results are shown as the mean ± SD.

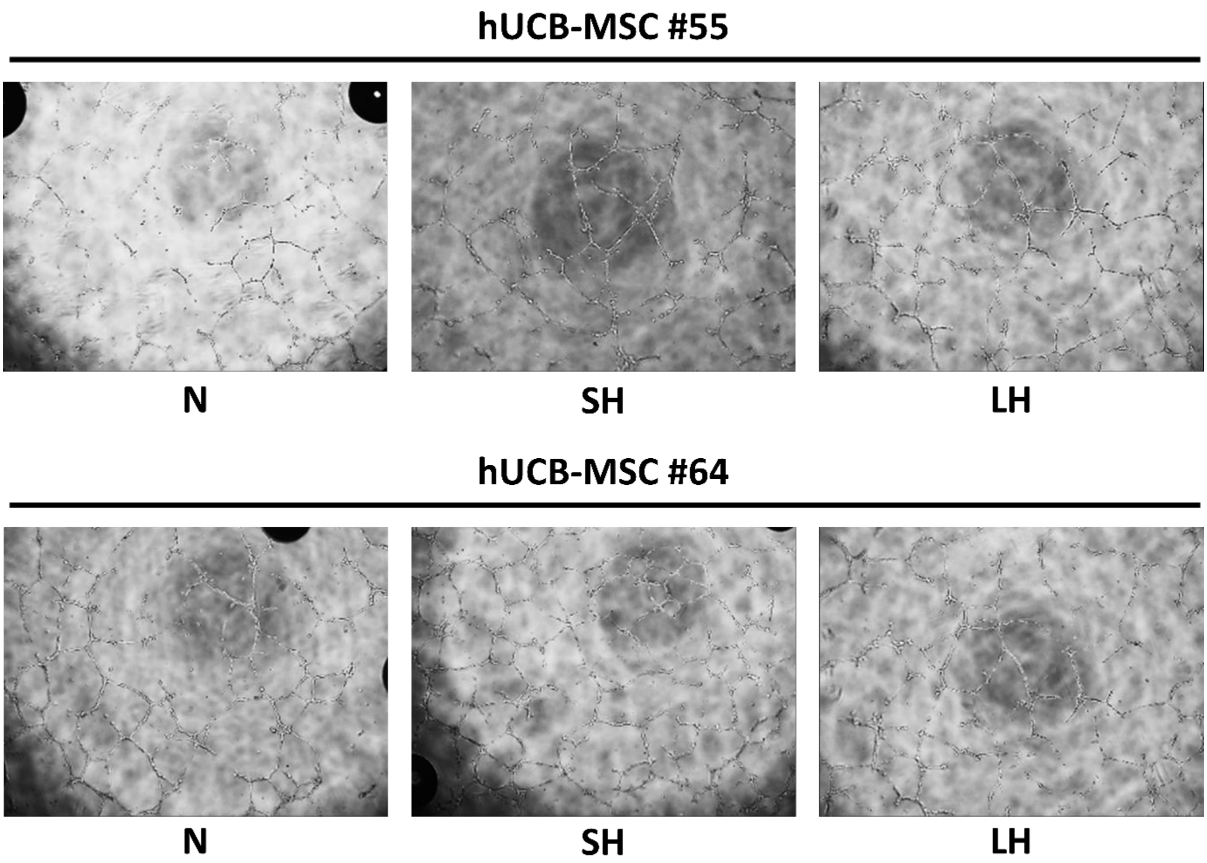

Supplementary Figure S3. Original images of capillary-like formation assay of hUCB-MSCs #55 and #64

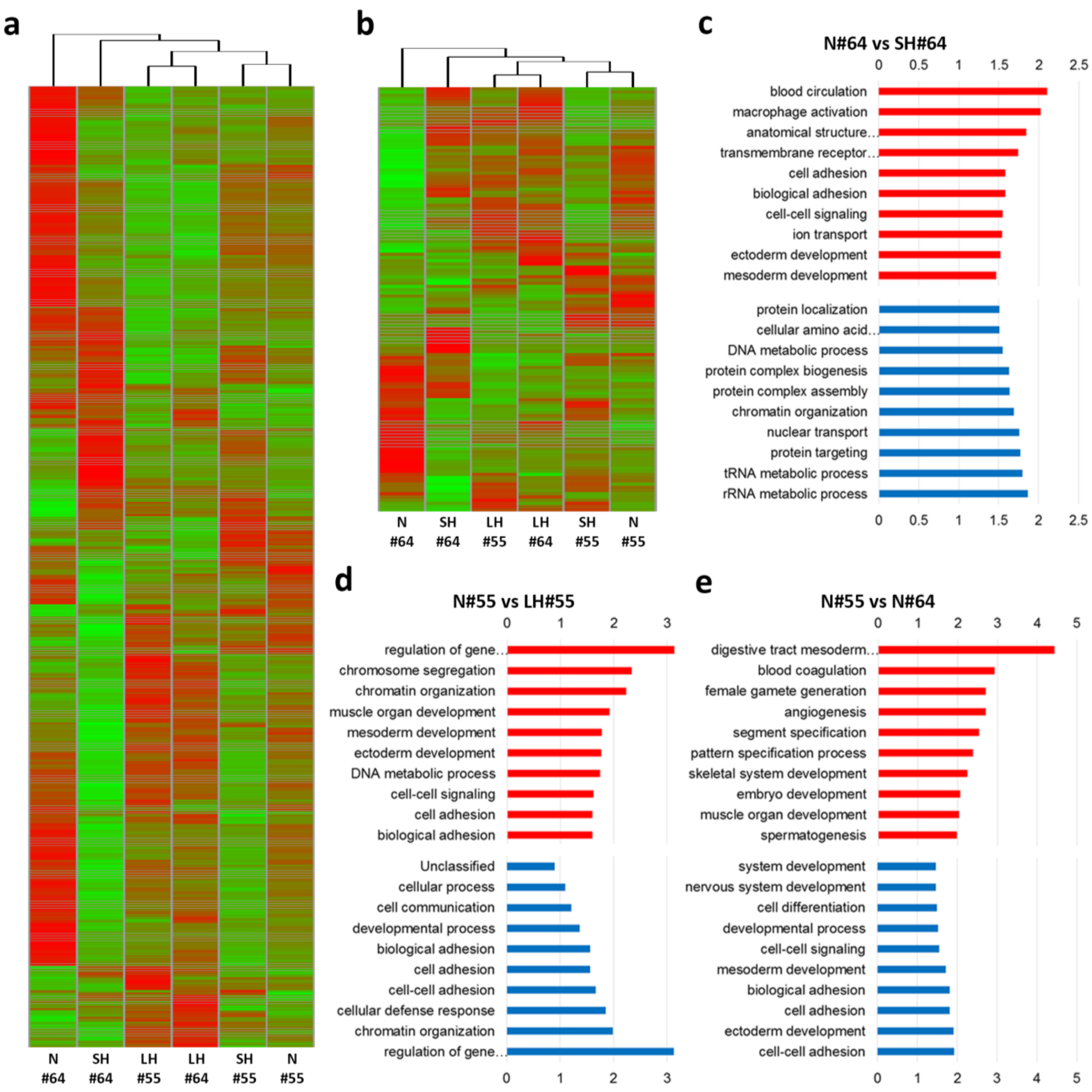

Supplementary Figure S4. Global gene expression analysis shows donor-dependent changes in gene expression patterns (a-b) The global genome heatmap with hierarchical cluster analysis shows similarities of genes expressed at an expression level of more than 100 (a) and genes related to the HIF-1 and VEGF signaling pathways (b). (c-e) GO function enrichment analysis indicated the top 10 categories related to the up-regulated genes (red) and the down-regulated genes (blue) in N#64 versus SH#64 (c), N#55 versus LH#55 (d) and N#55 versus N#64 (e). The data were analyzed using the PANTHER Classification System.

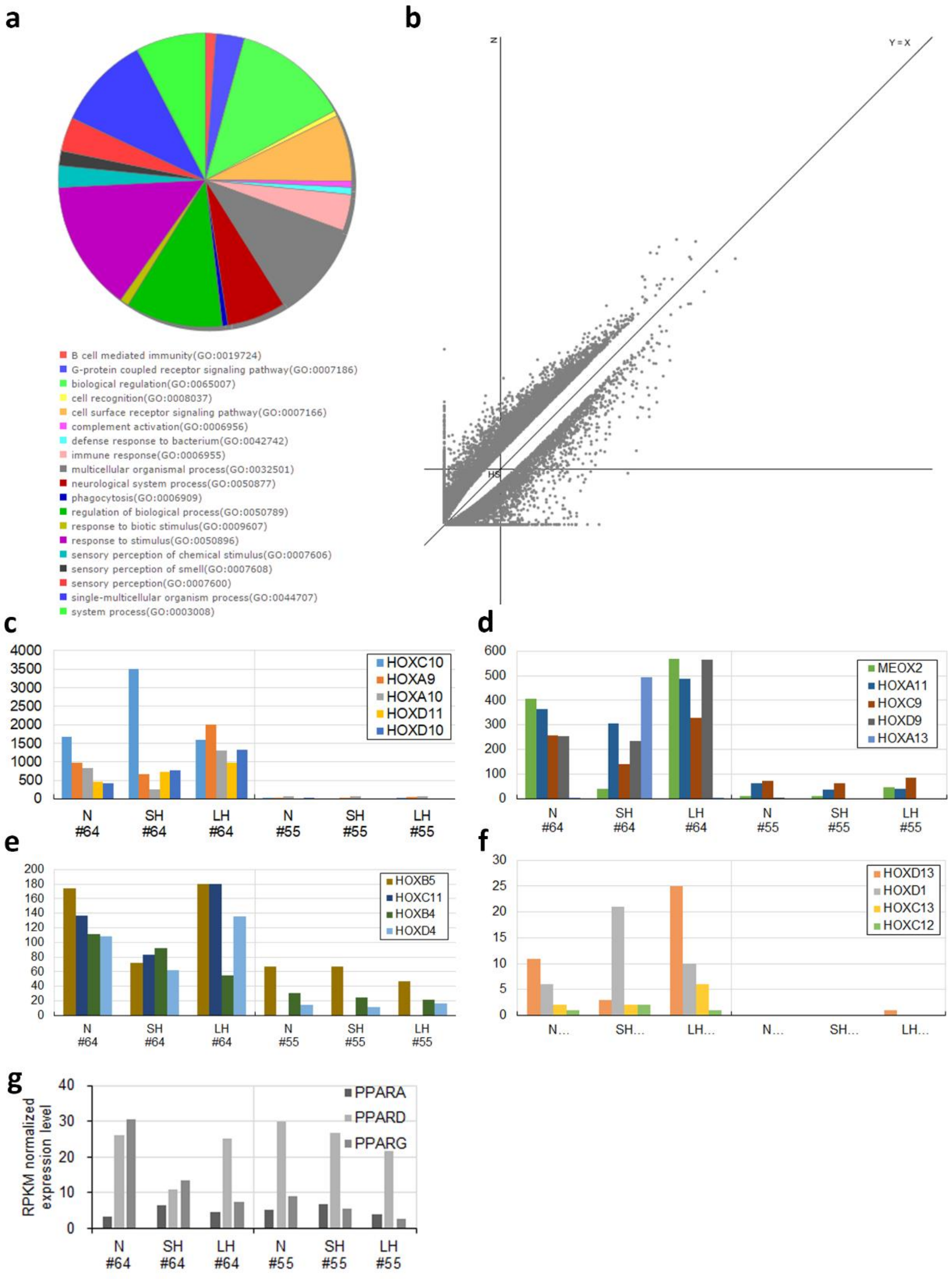

Supplementary Figure S5. mRNA expression profiling in hypoxic preconditioning hUCB-MSCs (a) The *PANTHER* analysis of biological processes of transcriptomes up- and down-regulated in the hypoxic preconditioning hUCB-MSC SH#64 compared to the N#64. (b) In this analysis, genes that showed a more than 2-fold alteration were illustrated as a scatter plot. (c-f) Normalized expression levels of genes that showed the highest fold enrichment in the category "DNA binding protein" between two non-preconditioning hUCB-MSCs N#64 and N#55 were revealed. (g) The mRNA sequencing analysis was indicated normalized gene expression changes of angiogenic receptors (PPARA, PPARD, PPARG) in both hUCB-MSC lines #64 and #55 as compared to the hypoxic preconditioned SH- or LH#64 and SH- or LH#55.

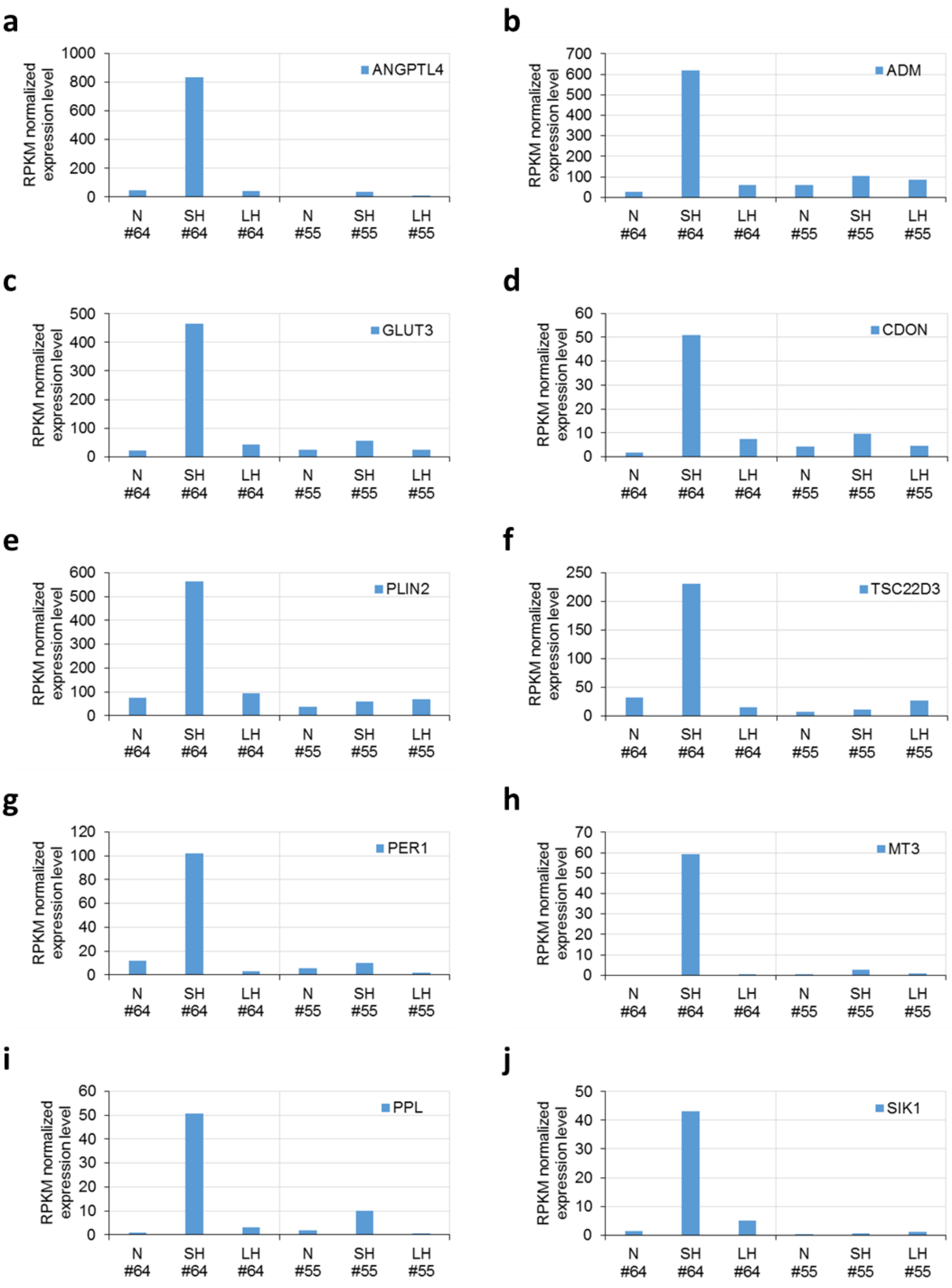

Supplementary Figure S6. 10 genes selected form NGS analysis Of entire gene expression data from the NGS analysis, 10 genes expressing at a more than 5-fold higher level in hypoxia condition SH#64, compared to any other samples N#55, SH#55, LH#55 and LH#64 was selected (a) ANGPTL4 (b) ADM (c) CDON (d) GLUT3 (e) PLIN2 (f) TSC22D3 (g) PER1 (h) MT3 (i) PPL (j) SIK1.
